# Job Dissatisfaction Mediated the Associations Between Work Stress and Mental Health Problems

**DOI:** 10.3389/fpsyt.2021.711263

**Published:** 2021-09-16

**Authors:** Dan Qiu, Ruiqi Li, Yilu Li, Jun He, Feiyun Ouyang, Dan Luo, Shuiyuan Xiao

**Affiliations:** ^1^Department of Social Medicine and Health Management, Xiangya School of Public Health, Central South University, Changsha, China; ^2^Lixia Center for Disease Control and Prevention of Jinan, Jinan, China; ^3^Mental Health Institute, Second Xiangya Hospital, Central South University, Changsha, China

**Keywords:** work stress, job satisfaction, mental health, working adults, Chinese

## Abstract

**Objective:** This study aimed to explore the relationships and the underlying mechanisms between work stress and mental health problems, and potential mediation effects through job dissatisfaction in a working population.

**Methods:** A large population-based study among workers in China was conducted. The self-reported scales of assessing job dissatisfaction and work stress were included in the questionnaire. Generalized Anxiety Disorder-2 and Patient Health Questionnaire-2 were used for assessment of mental health. Univariate logistic regression was conducted to test the associations between work stress and mental health. Path analysis was conducted to test the proposed mediation model.

**Results:** Of the 6,190 included employees, 27.72% reported that they perceived work stress, 14.84% of them reported that they were not satisfied with their work, 5.01% of the employees reported depressive symptoms, and 3.75% of the employees reported anxiety symptoms. The results of univariate logistic regression showed that employees who perceived work stress were more likely to report anxiety symptoms (adjusted odds ratio (AOR) = 2.78; 95% CI: 2.03–3.79) or depressive symptoms (AOR = 1.61; 95% CI: 1.22–2.12). The path analysis showed that work stress was positively associated with job dissatisfaction. Job dissatisfaction mediated the relationship between work stress and mental health problems among Chinese working adults.

**Conclusion:** This study suggests the importance of psychosocial work environment for mental health among Chinese working adults. Work dissatisfaction is a stressor that may induce negative consequences on the mental health among Chinese workers. Interventions to help workers with stress management may be beneficial for their mental health.

## Introduction

Depression is a prevalent mental health problem in the working population worldwide. It is reported that the global point prevalence of depression in the working population ranged from 3.73 to 19.0% (1–3). For anxiety, another prevalent mental health problem across the world, the prevalence of anxiety among the working population ranged from 5.40 to 20.70% (4, 5). Previous studies have shown that mental health problems at work could reduce employees' work productivity and performance, could increase disability, could reduce quality of life, and may lead to premature early retirement, which results in direct social and economic costs (6–8).

As one of the most important health issues, stress is quite prevalent among the working population (9–11). Research has been conducted on the correlates of mental health and work stress. The available evidence suggests that mental health may be closely related to work stress in the general population (1, 8, 12). Employees who experienced some work-related stressors would be more likely to experience job dissatisfaction (10), and in turn, such dissatisfaction would be positively associated with mental health problems (13).

Over recent decades, the labor market in China has undergone significant changes. There has been a shift in the focus of occupational health from physical hazards in the workplace to the impact of the psychosocial work environment on health (14). Key psychosocial work environmental factors such as low supervisor support and poor work atmosphere have been identified as predictors of mental health problems (13, 15). Also, it is widely accepted that when employees perceived job dissatisfaction, it may precipitate the development of anxiety or depressive symptoms (16, 17). The concept of job satisfaction was defined as the feeling that an individual person has about his or her job. Job satisfaction is an important aspect for the mental health of employees (18). However, the role of job satisfaction between work stress and mental health problems among Chinese employees was unclear.

Currently, numerous studies have been conducted on the work-related factors associated with mental health problems of employees; very few studies focused on how work-related factors will affect their mental health (19). The underlying mechanisms of the relationship between work-related factors and mental problems among working adults in developing countries were unclear. In the current study, we firstly investigated the levels of two mental health problems (i.e., anxiety symptoms and depressive symptoms) in the Chinese employees and their associations with work stress. We further investigated the roles of job dissatisfaction as mediators of such associations by fitting a path analysis. This study elucidates the relationships and the underlying mechanisms between work stress and mental health problems using a serial multiple mediation model. Specifically, we hypothesized that 1) perceived work stress would be positively associated job satisfaction among Chinese working adults and that 2) job dissatisfaction would mediate the association between work stress and anxiety/depressive symptoms.

## Methods

### Ethics Statement

This study was approved by the Human Research Ethics Committee of Central South University. Written informed consent was obtained before interviews were conducted.

### Participants

This cross-sectional study was conducted between January 2018 and October 2019 in the Health Management Center of the Third Xiangya Hospital of Central South University, Changsha, China. Employees were consecutively recruited if they (a) were employees who worked in Changsha City and (b) were aged between 18 and 60 years.

### Procedure

This project aimed to investigate employees aged 18 to 60 years and lived in Changsha, Hunan Province. Data were collected from January 2018 to October 2019. A total of 10 companies or organizations that agreed to participate in our project were recruited. Once a company or organization has agreed to participate in our project, all employees in that company or organization will be invited to the Third Xiangya Hospital, to complete the questionnaire online, and to undergo health examinations. A digital self-reported questionnaire platform was established to collect information on employees' health status and covariates. Recruited employees accessed the questionnaire with URLs sent by Short Messaging Service (SMS) and answered the questions via cellphone, tablet, or PC. After written informed consent was obtained, employees were enrolled in the study and completed study questionnaires. In the current study, all the employees (*n* = 7,045) who worked in the 10 company or organizations were recruited, 635 of them refused to participate in our project, and 6,410 employees were enrolled in the study.

### Measures

#### Sociodemographic Characteristics

Background variables included gender, age, marital status (married or single/divorced/widowed), educational achievement (primary school, secondary school/university/university, or above), level of employment, and alcohol consumption (never/occasional drinking, former drinkers, or current drinkers), and smoking status (never/occasional, former smokers, or current smokers). In this study, alcohol use was defined as drinking alcohol at least once a week for at least half a year. Smoking is defined as smoking at least one cigarette a day for at least half a year.

#### Work Stress

We determined work stress by using a single item. Previous studies showed that correlation patterns and multivariate analysis revealed a strong and significant association between the single-item measure and the other scale analysis on work stress (20, 21). In this study, the outcome variable “work stress” was evaluated by self-report, with the response options consisting of “no” and “yes” on a Likert-type 2-point scale.

Participants were asked by the question “Generally, have you perceived work stress over the past year?” Participants rated the items on a Likert scale (0 = no to 1 = yes).

#### Job Dissatisfaction

We determined job dissatisfaction by using a single item. Previous studies showed that correlation patterns and multivariate analysis revealed a strong and significant association between the single-item measure and the other scale analysis on job satisfaction (22). In this study, the outcome variable “job dissatisfaction” was evaluated by self-report, with the response options consisting of “no” and “yes” on a Likert-type 2-point scale.

Participants were asked by the question “Generally, have you perceived dissatisfaction with your job over the past year?” Participants rated the items on a Likert scale (0 = no to 1 = yes).

#### Depressive Symptoms

The Patient Health Questionnaire-2 (PHQ-2), a short version of the Patient Health Questionnaire-9 (PHQ-9), is a two-item scale that was used to assess depressive symptoms (23). Each item in PHQ-2 asked about the frequency of a depressive symptom experienced over the past 2 weeks on a 4-point Likert scale ranging from 0 (never) to 3 (almost every day), and the total score is 6. The Chinese version of PHQ-2 was used in this study, with a higher score indicating greater depressive symptoms (24). Previous research demonstrated that PHQ-2 has acceptable properties for identifying depressive symptoms among the Chinese population (23, 24). The scale had high internal consistency in this study (Cronbach's alpha = 0.81).

#### Anxiety Symptoms

The Generalized Anxiety Disorder-2 (GAD-2), a short version of the Generalized Anxiety Disorder-7 (GAD-7), is a two-item scale that was used to assess anxiety symptoms (25). Each item in GAD-2 asked about the frequency of an anxiety symptom experienced over the past 2 weeks on a 4-point Likert scale ranging from 0 (never) to 3 (almost every day), and the total score is 6. The Chinese version of GAD-2 was used in this study, with a higher score indicating greater anxiety symptoms. A previous study showed that GAD-2 has acceptable properties for identifying anxiety symptoms among the Chinese population (25). The scale had high internal consistency in this study (Cronbach's alpha = 0.85).

#### Analytic Plan

Descriptive statistics were computed for the independent (work stress and job dissatisfaction) and dependent variables (anxiety symptoms and depressive symptoms). Continuous variables of anxiety symptoms and depressive symptoms were used in our analyses (26). To test the associations between background variables and anxiety symptoms or depressive symptoms, univariate linear regression analyses were conducted. Dummy variables of the categorical background variables with more than two categories were created. Statistical significance level was set at *p* < 0.05. Those sociodemographic variables that were significantly associated with the dependent variables were controlled for in the path analysis. In addition, univariate logistic regression was performed to examine the association between variables (work stress and job dissatisfaction) and mental health problems (depressive symptoms and anxiety symptoms). After controlling the background characteristics, we included work stress and job dissatisfaction into a multivariate logistic regression model to obtain adjusted odds ratios (AORs) and the corresponding confidence intervals (CIs).

Pearson's correlation analysis was conducted to examine the associations among work stress, job dissatisfaction, anxiety symptoms, and depressive symptoms. Furthermore, the measurement model was tested using confirmatory factor analysis (CFA), which examined the goodness of fit of the pattern of observed indicators for the latent constructs in the proposed model. The hypothesized directionality of the relationships among the constructs and the overall fit of the model were examined in the path analysis. To test how well the model fitted the observed data, the χ^2^ test, the comparative fit index (CFI), the non-normed fit index (NNFI), and the root mean square error of approximation (RMSEA) were used. A non-significant *p*-value (*p* > 0.05) of the χ^2^ test represents an adequate model fit. CFI and NNFI values of larger than 0.90 and an RMSEA value < 0.08 indicate a reasonable model fit (27).

Standardized path coefficients were reported. Cohen's (1988) conventions to interpret effect size for the test were used (28), and Cohen's *f* ^2^ was reported; values of 0.02, 0.15, and 0.35 represent small, medium, and large effects, respectively (26). To identify the mediation effects of job dissatisfaction for the relationships between work stress and mental health problems, bootstrap procedure was used to test the mediation effect (29, 30). Following the recommendations of Shrout and Bolger (30), bias-corrected CIs, based on 5,000 resamples, were used in the bootstrap analysis. The 95% CIs of the indirect effects were obtained from 5,000 bootstrap resamples. A statistically significant mediation effect is observed when the CI does not include zero. The proportion mediated (PM) was used to evaluate the effect size of the mediation analysis. Descriptive statistics and univariate linear regression analyses were conducted by SPSS 26.0; model testing and mediation testing were conducted by AMOS 23.0.

## Results

### Descriptive Statistics

A total of 6,410 employees participated in this study. Completing the measurement of work stress, job satisfaction, mental health, and covariates is one of the inclusion criteria, with 6,190 participants available for the final analysis. The mean age of the sample was 38.68 years (SD = 9.25); most of them were younger than 40 years (58.77%), 56.04% of the sample were female, and 43.96% were male. More than half of the employees (57.5%) had a college education. Most of them (85.06%) were currently married or cohabitating with someone; 12.10% were single; 2.84% were divorced/separated/widowed. Of the 6,190 employees, 36.28% were primary employees (Table 1), 11.68% were current drinkers, and 12.18% were current smokers. Of the included employees, 27.72% reported that they perceived work stress, 14.84% of them reported that they were not satisfied with their work, 5.01% of the participants were classified as having probable depression, and 3.75% of the participants were classified as having anxiety symptoms. The means and standard deviations (SDs) of depressive symptoms and anxiety symptoms are displayed in Table 2.

**Table 1:** Descriptive and univariate regression analyses of background characteristics on mental health problems.

**Demographic characteristics**	**N/%**	**Depression**	**Anxiety**
		B (95% CI)	B (95% CI)
**Gender**			
Male	2,721 (43.96%)	Ref	Ref
Female	3,469 (56.04%)	0.18 (0.12–0.25)^***^	0.17 (0.11–0.23)^***^
**Age (years)**			
18~30	1,206 (19.48%)	Ref	Ref
31~40	2,432 (39.29%)	0.39 (0.27–0.51)^***^	0.32 (0.21, 0.43)^***^
41 ~ 50	1,584 (25.59%)	0.40 (0.30–0.48)^***^	0.37 (0.28, 0.45)^***^
51 ~ 60	968 (15.64%)	0.17 (0.08–0.26)^***^	0.17 (0.09, 0.25)^***^
**Education**			
High school or below	319 (5.15%)	Ref	Ref
University	3,558 (57.48%)	0.13 (0.04, 0.22)^*^	0.08 (0.00, 0.17)
Graduate	2,313 (37.37%)	0.03 (−0.12, 0.19)	−0.05 (−0.20, 0.10)
**Marital status**			
Divorced/widowed	176 (2.84%)	Ref	Ref
Single	749 (12.10%)	−0.19 (−0.32, −0.06)^**^	−0.13 (−0.26, −0.01)^*^
Married	5,265 (85.06%)	−0.08 (−0.14, −0.02)^**^	−0.01 (−0.07, −0.04)
**Grades of employment**			
Primary	2,246 (36.28%)	Ref	Ref
Intermediate	2,554 (41.26%)	−0.01 (−0.09, 0.09)	−0.05 (−0.14, 0.03)
Senior/deputy senior	1,390 (22.46%)	0.05 (−0.02, 0.12)	0.03 (−0.03, 0.10)
**Drinking status**			
Never drink	5,365 (86.67%)	Ref	Ref
Current drinkers	723 (11.68%)	0.08 (−0.01, 0.17)	0.02 (−0.06, 0.11)
Ex-drinkers	102 (1.65%)	−0.10 (−0.32, 0.11)	0.01 (−0.19, 0.21)
**Smoking status**			
Never smoke	5,369 (86.74%)	Ref	Ref
Current smokers	754 (12.18%)	0.12 (0.03, 0.21)^**^	0.13 (0.05, 0.22)^**^
Ex-smokers	67 (1.08%)	0.37 (0.10, 0.63)^**^	0.17 (−0.07, 0.41)

*CI, confidence interval*.

* *p < 0.05*,

** *p < 0.01*

*** *p < 0.001*.

**Table 2:** Logistic regression analysis of related variables on mental health problems.

	**Depression**		**Anxiety**	
	**OR_**C**_**	**OR_**A**_**	**OR_**C**_**	**OR_**A**_**
**Work stress**			
No	Ref		Ref	
Yes	2.80 (2.22–3.53)^**^	1.61 (1.22–2.12)^**^	4.39 (3.35–5.75)^**^	2.78 (2.03–3.79)^**^
**Job dissatisfaction**			
No	Ref		Ref	
Yes	4.72 (3.73–5.99)^**^	2.94 (2.19–3.95)^**^	4.58 (3.49–5.99)^**^	2.19 (1.57–3.03)^**^

*ORu, univariate odds ratio; AOR, adjusted odds ratio for background characteristics included in this study; CI, confidence interval*.

* * p < 0.05*,

** * p < 0.01*.

### Comparisons on the Key Variables by the Sociodemographics

As presented in Table 1, female sex, older age, marital status as single, higher education levels, and smoking were significantly associated with higher depressive symptoms (*p* < 0.05). Female sex, older age, higher education levels, and smoking were significantly associated with higher anxiety symptoms (*p* < 0.05). These demographic variables that significantly associated with the dependent variables were included in the path analysis.

The adjusted regression results showed that work stress (AOR = 1.61; 95% CI: 1.22–2.12) and job dissatisfaction (AOR = 2.94; 95% CI: 2.19–3.95) were all positively associated with depressive symptoms (*p* < 0.001) (Table 2). Also, work stress (AOR = 2.78; 95% CI: 2.03–3.79) and job dissatisfaction (AOR = 2.19; 95% CI: 1.57–3.03) were all positively associated with anxiety symptoms (*p* < 0.001).

### Correlations Among the Variables

Pearson's correlation analysis (Table 3) showed that work stress was positively correlated with job dissatisfaction (*r* = 0.25 and 0.40) (*p* < 0.01). Work stress was also significantly correlated with anxiety symptoms (*r* = 0.27, *p* < 0.01) and depressive symptoms (*r* = 0.31, *p* < 0.01). Job dissatisfaction was significantly correlated with anxiety symptoms and depressive symptoms (*r* = 0.30 and 0.27) (*p* < 0.01).

**Table 3:** Pearson's correlation.

	**Mean ± SD**	**1**	**2**	**4**
1. Work stress	0.28 **±** 0.45			
2. Job dissatisfaction	0.15 **±** 0.35	0.40^*^		
3. Depression	0.67 **±** 1.02	0.27^*^	0.30^*^	
4. Anxiety	0.55 **±** 0.93	0.31^*^	0.27^*^	0.75^*^

* *p < 0.01*.

### Model Testing

**Figure 1:**
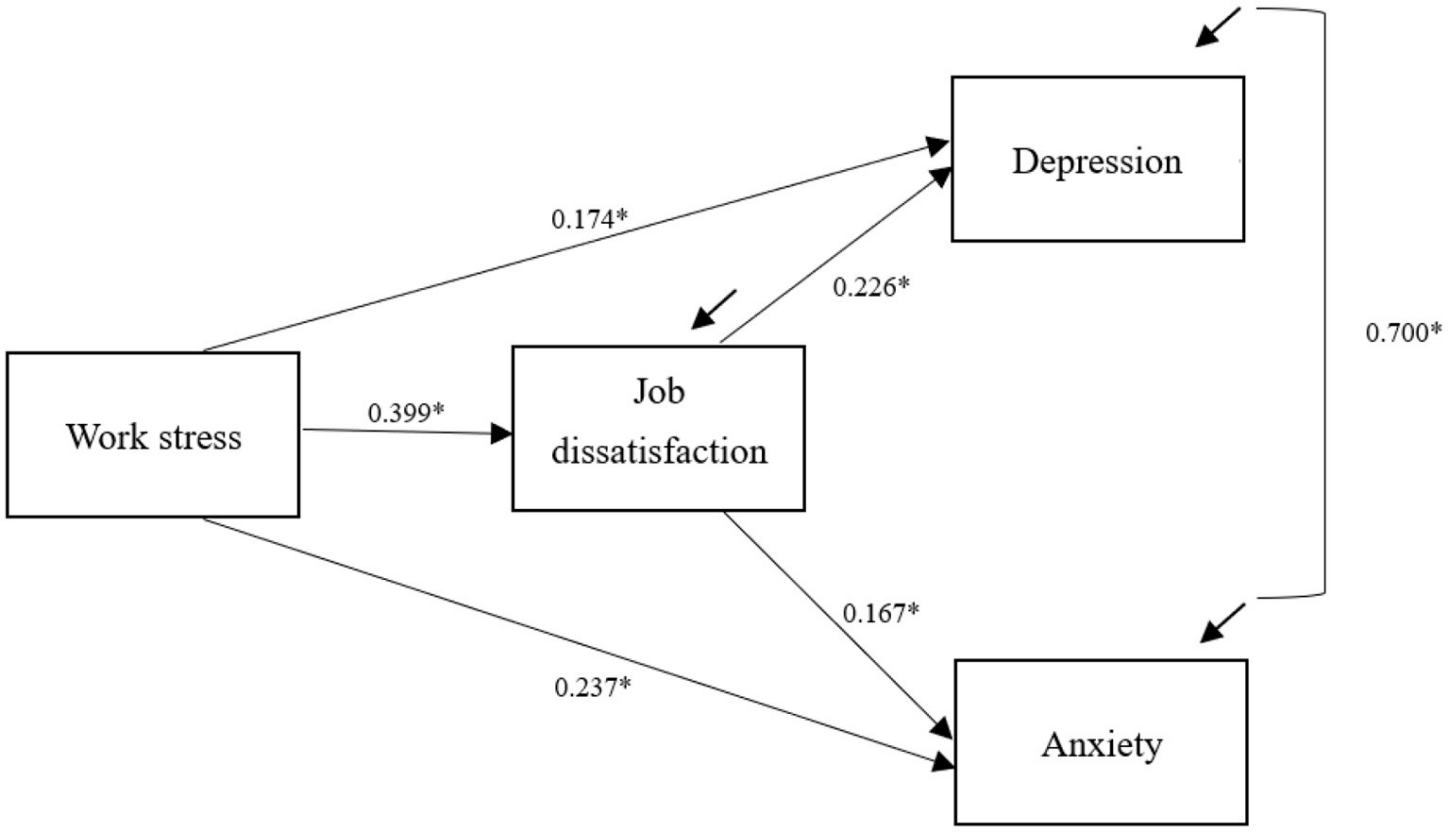
The proposed model with standardized coefficients. *p < 0.05. For simplicity reasons, the demographic variables controlled in the model are not presented in the figure. Gender was significantly associated with depression (β = 0.08, *p* < 0.01) and anxiety (β = 0.08, *p* < 0.01). Age was significantly associated with depression (β = −0.09, *p* < 0.05) and anxiety (β = −0.07, *p* < 0.01). Education level was significantly associated with depression (β = −0.05, *p* < 0.01). Smoking was significantly associated with depression (β = 0.04, *p* < 0.01) and anxiety (β = 0.04, *p* < 0.01).

Verification analysis of the structural equation model proved that the model fit the data well (Figure 1), χ^2^(16) = 291.179, *p* < 0.05, CFI = 0.97, NNFI = 0.91, RMSEA = 0.06. All factor loadings were significant at *p* < 0.001.

### Path Coefficients

As hypothesized, the direct path from work stress to depressive symptoms (B = 0.43, β = 0.17, *p* < 0.001, Cohen's *f* ^2^ = 0.03) and anxiety symptoms (B = 0.54, β = 0.24, *p* < 0.001, Cohen's *f* ^2^ = 0.06) was significant and positive, respectively. A higher level of work stress was associated with a lower level of job satisfaction (B = 0.40, β = 0.32, *p* < 0.001, Cohen's *f* ^2^ = 0.11). Low job satisfaction (B = 0.48, β = 0.17, *p* < 0.001, Cohen's *f* ^2^ = 0.03) was significantly associated with anxiety symptoms. Also, job dissatisfaction (B = 0.70, β = 0.23, *p* < 0.001, Cohen's *f* ^2^ = 0.06) was significantly associated with depressive symptoms. Anxiety symptoms were positively associated with depressive symptoms (*p* < 0.001).

### Mediation Effects

Bootstrapping analyses indicated that work stress was indirectly associated with depressive symptoms through job dissatisfaction (B = 0.22, β = 0.09, *p* < 0.001, PM = 15.85%; 95% CI: 0.08–0.11). Further analyses showed that the individual mediation effect of job dissatisfaction (*p* < 0.001) for the associations between work stress and depressive symptoms was statistically significant. Also, work stress was indirectly associated with anxiety symptoms through job dissatisfaction (B = 0.15, β = 0.07, *p* < 0.001, PM = 12.31%; 95% CI: 0.05–0.08). Further analyses showed that the individual mediation effect of job dissatisfaction (*p* < 0.001) for the associations between work stress and anxiety symptoms was statistically significant. See Table 4 for the details.

**Table 4:** Bootstrap analyses of total, direct, and indirect effects of the mediation model.

**Paths**	**β**	**Boot *SE***	**95%*CI^a^***	***P***
**Direct effect**				
Work stress → depression	0.17	0.014	0.14-0.20	*p* <0.01
Work stress → anxiety	0.24	0.014	0.21-0.26	*p* <0.01
**Indirect effect**				
Work stress → job dissatisfaction → depression	0.09	0.007	0.07-0.11	*p* <0.01
Work stress → job dissatisfaction → anxiety	0.07	0.007	0.05-0.08	*p* <0.01
**Total effect**	0.57	0.025	0.52-0.62	*p* <0.01

a *Bias-corrected percentile method was presented based on 5,000 bootstraps samples*.

## Discussion

To our best knowledge, this is the first study that proposed and tested the potential mediation effects of job dissatisfaction on the association between work stress and mental health problems among Chinese employees. The findings in the current study generally support the application of Jeffrey Johnson and Tores Theorell's “demand-control-support” model (14, 31) among Chinese working adults. The two overall hypotheses were confirmed. Working adults who had anxiety and depressive symptoms were more likely to report perceived work stress. In addition, work stress was positively associated with job dissatisfaction. Job dissatisfaction mediated the relationship between work stress and mental health problems among working adults in a Chinese culture.

Chinese working adults who were older and single were found to have higher levels of mental health problems, which was consistent with other studies (5, 32). This vulnerable population may benefit from primary prevention and support services. Some group-based psychosocial interventions were able to enhance mental health among working adults by addressing issues related to psychosocial work environment (33). Caution should be taken regarding cultural sensitivity when developing such interventions, as the Chinese may perceive psychosocial work stressors differently. Some cultural barriers may also discourage related help-seeking behaviors (34, 35); future research is needed to discern such cultural differences. The multivariate relationship between psychosocial work-related variables, work stress, and mental health-related variables was statistically significant, showing that psychosocial work environmental issues are important in understanding Chinese working adults' mental health. In the results of path analysis, it was demonstrated that work stress would directly impact mental health problems, which was consistent with studies conducted in other countries (36).

Based on the “demand-control-support” model, employees who reported low job satisfaction may mean that the workers' decision latitude in the task is low and that they lack resources to deal with demands (14). In this case, employees easily perceived work stress and were more likely to report health problems (14). Supporting our hypothesis, we found a strong mediating effect of job dissatisfaction on the association between work stress and mental health problems (15.85% on depressive symptoms and 12.31% on anxiety symptoms). These associations between these variables were also similar to the studies of Jaradat et al. (37–39). The results of path analysis in our study indicated that work stress would directly impact job dissatisfaction, which would in turn directly influence mental health among Chinese adults. The mediating effect of job dissatisfaction may explain the underlying mechanisms between psychosocial work environment and mental health problems among Chinese working adults to a certain extent. Some psychosocial interventions (such as cognitive behavior therapy and mindfulness-based stress reduction) were able to enhance mental health among working adults by addressing issues related to psychosocial work environment and job dissatisfaction. Although the situation is changing in recent years, some countries (such as China and South Korea) in Asia are relatively partial to authoritarianism, collectivism, and kinship, which are different from the horizontal organization and rationalism that are popular in Western countries (12). The Chinese workers are hardworking and obedient due to the social culture of nationalism, stability, and harmony. Thus, the negative impact of poor psychosocial work environment among Chinese workers was particularly significant. We think it is necessary to study the influence of social culture on stress management and mental health among the working population in developing countries.

The model of this study has some potential public health implications, as it demonstrated potential impact of work stress on mental health problems and the mediation of the relationship via job dissatisfaction. The results highlight a potential need to improve job satisfaction for working adults in China, especially for those female employees who reported work stress, as job dissatisfaction may be a significant social issue for these groups, and it can induce mental health problems. Other potential consequences and associates of job dissatisfaction in addition to mental health problems also need to be tested in this sample. Moreover, working requirements of employees are complex; we think future studies need to explore the potential moderators in the relationships between psychosocial work factors and mental health problems. China has experienced rapid social and economic changes over the past 30 years, and there was a considerable change in the workforce structure. Employees are commonly faced with greater demands, less job security, and higher imbalance between work and family. These situations are likely to be stressful; thus, psychological disorders may increasingly be caused by work-related stressors (40). The job strain model (demand-control-support model) has already been confirmed in a large number of studies on employees in developed countries, and many psychosocial work factors have been linked to mental health problems (4, 32). Recently, studies have suggested that other exposures may play a role in mental disorders (such as effort–reward imbalance and workplace bullying) (41). Hence, it is necessary to explore the impact of psychosocial work environment on mental health among employees in developing countries more comprehensively.

In interpreting the present results, it is important to note some limitations. First, this study was a cross-sectional design; we think that longitudinal studies are needed to confirm a temporal effect of these associations. Second, this study did not include employees who worked in rural areas, or those unemployed or individuals with precarious jobs; thus, it is not representative of the general population of working age, which may limit the generalizability of our findings. Third, we assessed depressive/anxiety symptoms rather than clinical depression or anxiety, even though it has been suggested that significant mental health symptomatology is a risk factor for clinical mental health disorder (42). Thus, we cannot completely exclude misclassification bias as a source of error that explains the current observed associations. A previous study suggests that responses to a single-item measure of work stress and job satisfaction may provide a useful shorthand indication of likely exposure to problematic psychosocial working environment and impaired health (21, 22). That being the case, such a single-item measure has potential application in organizational psychosocial environment management programs (20). In order to support the application of a single-item measure of work stress and job satisfaction in such programs, it is important to first establish its construct validity (21). However, the data generated by a single-item measure provide little insight into the private psychological processes that take place to arrive at a publicly disclosed rating (21). This means that construct validity of our measurement on work stress and job satisfaction remains uncertain. Thus, we think that multi-item measures are needed in the future for work stress and job satisfaction among the current population. Finally, although we adjusted for a number of potential confounders, the results in this study might still be affected by unmeasured or residual confounding.

## Conclusion

Our study found significantly positive associations between work stress and mental health problems in both bivariate correlation analyses and path analysis. Together with the positive relationships between work stress and job dissatisfaction, our results support the negative role of psychosocial work-related factors on one's mental health and imply a potential need to improve psychosocial work environment through individual- or organizational-level interventions.

## Data Availability Statement

The raw data supporting the conclusions of this article will be made available by the authors, without undue reservation.

## Ethics Statement

The studies involving human participants were reviewed and approved by the Human Research Ethics Committee of Central South University. The patients/participants provided their written informed consent to participate in this study.

## Author Contributions

DQ and SX: study design. DQ, YL, RL, JH, DL, and FO: data collection. DQ, RL, and SX: data analysis and interpretation. All authors were involved in writing the paper and had final approval of the submitted and published versions.

## Funding

This research was supported by the Ministry of Science and Technology of China (grant no. 2016YFC0900802). The funding agency did not take part in the design of the study and collection, analysis, and interpretation of data and in writing the manuscript.

## Conflict of Interest

The authors declare that the research was conducted in the absence of any commercial or financial relationships that could be construed as a potential conflict of interest.

## Publisher's Note

All claims expressed in this article are solely those of the authors and do not necessarily represent those of their affiliated organizations, or those of the publisher, the editors and the reviewers. Any product that may be evaluated in this article, or claim that may be made by its manufacturer, is not guaranteed or endorsed by the publisher.

## Abbreviations

PHQ-2: patient health questionnaire-2
PHQ-9: patient health questionnaire-9
CI: confidence interval.
